# NMR Untargeted and HPLC-MS/MS Targeted Metabolomic Approaches for Evaluating Styrene Exposure in the Urine of Shipyard Workers

**DOI:** 10.3390/toxics12030182

**Published:** 2024-02-28

**Authors:** Ottavia Giampaoli, Fabio Sciubba, Giovanna Tranfo, Renata Sisto, Daniela Pigini, Michele De Rosa, Adriano Patriarca, Alfredo Miccheli, Anna Rita Fetoni, Laura Tricarico, Mariangela Spagnoli

**Affiliations:** 1 NMR-Based Metabolomics Laboratory (NMLab), Sapienza University of Rome, Piazzale Aldo Moro 5, 00185 Rome, Italy; ottavia.giampaoli@uniroma1.it (O.G.); fabio.sciubba@uniroma1.it (F.S.); a.miccheli@gmail.com (A.M.); 2 Department of Environmental Biology, Sapienza University of Rome, Piazzale Aldo Moro 5, 00185 Rome, Italy; 3 Department of Occupational and Environmental Medicine, Epidemiology and Hygiene, INAIL, Via Fontana Candida 1, 00078 Monte Porzio Catone, Italy; g.tranfo@inail.it (G.T.); r.sisto@inail.it (R.S.); d.pigini@inail.it (D.P.); 4 Department of Chemistry, Sapienza University of Rome, Piazzale Aldo Moro 5, 00185 Rome, Italy; michele.derosa@uniroma1.it (M.D.R.); adriano.patriarca@uniroma1.it (A.P.); 5 Department of Neuroscience, Reproductive and Odontostomatological Sciences-Audiology Section, University of Naples Federico II, 80131 Naples, Italy; annarita.fetoni@unina.it; 6 Faculty of Medicine and Surgery, Catholic University of the Sacred Hearth, Largo Agostino Gemelli 8, 00168 Rome, Italy; lauratricarico90bis@gmail.com

**Keywords:** styrene exposure, NMR-based metabolomics, oxidative stress biomarkers, urinary metabolic profiles

## Abstract

Due to its chemical properties, styrene is largely employed in the manufacturing of several products including rubber, polymers and resins, and it is particularly suitable for shipbuilding industry purposes. In this context, the main exposure to styrene occurs in occupational settings. Despite its widespread use, its long-term effects on human health at the occupational level are still unclear. The aim of this pilot study was to evaluate changes in styrene exposure biomarkers related to the metabolic and oxidative stress profiles in the urine of seventeen shipyard workers and seventeen non-exposed subjects. Urinary metabolites were assessed by means of NMR spectroscopy, including mandelic and phenylglyoxylic acids; four oxidative stress biomarkers, namely 8-oxo-7,8-dihydroguanine, 8-oxo-7,8-dihydroguanosine, and 8-oxo-7,8-dihydro-2′-deoxyguanosine and 3-nitrotyrosine, were evaluated via HPLC-MS/MS. The metabolic profiles of exposed workers showed both long- and short-term metabolic responses to styrene exposure compared to non-exposed subjects. From the comparison between non-exposed and before-shift workers, only 8-oxo-7,8-dihydroguanine and 8-oxo-7,8-dihydro-2′-deoxyguanosine levels were significantly different (long term exposure response). At the same time, comparing the non-exposed group with after-shift workers, we observed lower levels of pseudouridine and 1-methylnicotinamide and higher glutamine levels in after-shift workers. The comparison between before-shift and after-shift workers showed that 8-oxo-7,8-dihydroguanine significantly increased after the shift, suggesting its involvement in the exposure to styrene (short-term exposure response). The obtained results, although preliminary, allow us to lay the basis for further human studies aimed at establishing a global understanding of styrene metabolism.

## 1. Introduction

Since 1940, styrene has become an extremely important chemical employed in the manufacture of numerous products, starting from the synthesis of rubber [1]. Styrene is an aromatic hydrocarbon, characterized by a high chemical reactivity due to the double vinyl bond and, for these reasons, it is involved in the manufacture of polymers and resins [2,3]. During the thermoplastic formation process, exposure can occur due to thermal degradation induced by heating, resulting in the emission of fumes containing styrene monomers. Styrene appears as a colorless oily liquid, with a boiling point of 145.2 °C and a vapor pressure of 4.5 mmHg at 20 °C. Since the resin manufacturing process may involve high temperatures (above 200 °C), quantities of styrene monomer may be emitted into the surrounding environment [1].

The most substantial human exposure to styrene occurs in occupational settings [1]. Styrene can be absorbed through both inhalation and dermal contact; however, in a work environment, inhalation is the main route of absorption [4]. Subsequently, styrene is absorbed into the blood and distributed throughout the body. Styrene monomers are central nervous system (CNS) depressants with anesthesia-like properties and acute effects such as tiredness, impaired vision, headaches, impaired attention, and hearing deficits [5]. The International Agency for Research on Cancer (IARC) has included styrene in Group 2B, being possibly carcinogenic to humans. However, the long-term effects on human health of styrene exposure at occupational levels still remain unclear [6]. It has been estimated that only 8% of absorbed styrene is retained in adipose tissue, the half-life of which amounts to 2.8–5.2 days [7]. It has been shown in animal models that styrene is both hepatotoxic and pneumotoxic [8,9]; however, the precise mechanism by which styrene exerts it effects on the liver and lungs in humans is still unknown. These adverse effects have been considered to be related to the metabolism of styrene oxide [9]. Based on in vivo studies, styrene is oxidized by cytochrome P450-dependent monooxygenases (CYP450) situated predominantly in the liver to styrene oxide. Toxicological studies have shown both mutagenic and carcinogenic effects of styrene oxide, due to the fact that it is effective binding agent of DNA and DNA nucleobases [10,11].

Furthermore, styrene oxide conjugates with glutathione, decreasing its concentration in the liver and lungs. This leads to an increase in susceptibility to oxidative stress [12,13]. An indication of the oxidatively generated damage on DNA and RNA in humans is given by the quantification of the urinary levels of 8-oxo-7,8-dihydro-2′-deoxyguanosine (8-oxodGuo), 8-oxo-7,8-dihydroguanosine (8-oxoGuo), and 8-oxo-7,8-dihydroguanine (8-oxoGua), which are generated by an attack of the •OH radical on guanine, the main oxidation target, as it has the lowest redox potential among nucleobases [14,15]. As also observed in a recent work, these biomarkers are affected by styrene exposure in shipbuilding workers [16].

Furthermore, peroxynitrite (ONOO) can be generated both by nitrogen and oxygen reactive species, and can react with tyrosine in proteins or in free form to produce 3-NO_2_-tyrosine, a specific marker for oxidatively generated damage to proteins [17].

Styrene oxide is hydrated into phenylethanediol and finally oxidized into mandelic (MA) and phenylglyoxylic (PGA) acids [18], which are excreted in urine and are the urinary biomarkers of styrene [19].

The American Conference of Governmental Industrial Hygienists (ACGIH) established the biological exposure indices (BEI) related to the sum of the mandelic acid and phenylglyoxylic acid concentrations in urine, which are the concentrations that are most likely to be observed in specimens collected from healthy workers who have been exposed to chemicals to the same extent as workers with inhalation exposure at the threshold limit value–time weighted average (TLV–TWA) [20].

Metabolomics is defined as “the quantitative measurement of the dynamic multiparametric metabolic response of living systems to pathophysiological stimuli or genetic modification” [21] and represents an attractive molecular profiling technology [22]. Recently, the concepts of metabolomics and exposomics have been combined together to create a synergistic tool to more deeply understand the metabolic effect of occupational exposure to risk factors [23].

Walker et al. used plasma metabolomics to link occupational exposure to trichloroethylene to modifications to the metabolism, identifying, among other things, changes in the purine, methionine and cysteine metabolism following exposure [24]. Carter et al. found seven metabolites that differentiated exposed from unexposed workers, involving changes in beta-alanine metabolism, histidine metabolism, and glycine, serine, and threonine metabolism [25].

Xenobiotic metabolism and utilization involve many different enzymes, multiple organs, several compartments and even the microbiome, and are not always possible to screen for all possible genetic or tissue variants. Meanwhile, more sensitive markers are urgently needed, particularly for the early detection of environmental and occupational exposure effects [26].

In previous studies, the concentrations of styrene and its metabolites have been assessed in biological matrices by means of chromatographic techniques coupled with ultraviolet detection or mass spectrometry [27,28,29].

Nuclear magnetic resonance (NMR) has recently been gaining attention as a novel analytical platform for assessing the metabolome of biological matrices. Despite its low sensitivity compared to MS, NMR exhibits unique characteristics that are beneficial for metabolomics studies, such as its high reproducibility, selectivity and absolute quantitation of metabolites using a single internal standard [30]. In addition, NMR spectroscopy is the most powerful analytical tool for molecular structure identification [31], and in this context, could be a strongly reliable technique for assessing styrene metabolites in complex matrices, such as urine.

Given the complementarity of these two techniques, in this pilot study, we present for the first time a metabolomic multiplatform approach for investigating the urinary metabolic profile of shipyard workers exposed to styrene vapors. This study is based on a previously published work by Pigini et al., where two oxidative stress biomarkers, 8-oxoGua and 8-oxodGuo, were found to be higher in the urine of workers after the working shift and compared to controls, and where 8-oxoGuo was positively correlated in styrene-exposed workers with MA, PGA, and ∑MA + PGA [16].

Therefore, our aim is to relate changes in styrene dose biomarkers to the urinary metabolic profile and to the biomarkers of oxidatively generated damage, highlighting the short- and long-term effects that exposure to styrene far below the TLV–TWA could cause.

Thirty-five urinary metabolites of different chemical classes were assessed by means of NMR spectroscopy, as well as four oxidative stress metabolites, 8-oxoGua, 8-oxoGuo, 8-oxodGuo and 3-NO_2_Tyr, assessed via HPLC-MS/MS.

## 2. Materials and Methods

### 2.1. Study Design

The subjects included in this study, aged between 32 and 58 years old (mean age 45.8 ± 7.1), were seventeen male workers exposed to styrene during their occupational activities involving the assembly of fiberglass-reinforced plastic components of motorboats and seventeen healthy volunteers (mean age 59.4 ± 4.8) not exposed to styrene. Urine samples were collected from workers at the beginning of the working shift (BS) after fasting, and after their eight-hour work shift (AS). Characteristics of the subjects enrolled in this study are reported in Table 1.

In this pilot study, we firstly compared the healthy volunteers (C) with the workers at BS in order to evaluate metabolic changes due to long-term exposure, since samples were collected in the middle of the working week to avoid the clearance of xenobiotics during the weekend.

For the evaluation of eight-hour exposure (short-term effects), we compared C with AS workers, as well as BS and AS workers.

All of the shipyard workers wore the appropriate personal protective equipment, and the styrene dose biomarkers were below the exposure limits, as reported by BEI’s ACGIH, with the exception of one subject at AS.

This study was approved by the Ethical Committee of Fondazione Policlinico Universitario Agostino Gemelli, Università Cattolica del Sacro Cuore, protocol ID: 5117 (no-profit study), 3 August 2022. Written informed consent was obtained from all of the involved subjects.

The collection of urine samples was performed on the same day and each sample was transferred in duplicate, for NMR and HPLC-MS/MS analyses, into sterile polypropylene containers and stored at −80 °C.

### 2.2. Urine Preparation for NMR Analysis

The sample (1200 μL) was centrifuged at 4 °C for 15 min at 11,000× *g* to remove cells.

An aliquot of 100 μL of an internal standard solution of trimethylsilylpropionic acid-2,2,3,3-d4 acid (TSP) in D_2_O was added to 1 mL of the supernatant, for a final TSP concentration of 2 mM. Small amounts of NaOH or HCl were added to reach a final pH = 7, and then 700 μL of the sample was transferred into the NMR precision tube for the analysis.

### 2.3. ^1^H-NMR Spectroscopy for Urinary Metabolic Profile Assessment

A JEOL JNM-ECZR spectrometer (JEOL Ltd., Tokyo, Japan), equipped with a magnet operating at 14.09 Tesla and 600.17 MHz for the ^1^H working frequency, was used to record NMR spectra with the following setting parameters: temperature of 298 K, 64 k points and 64 scans, spectral width at 9.03 kHz (15 ppm), presaturation pulse length of 2.00 s, relaxation delay of 5.72 s, and an acquisition time of 5.81 s.

The identification of metabolites was conducted through bidimensional experiments (^1^H-^1^H TOCSY and ^1^H-^13^C HSQC) applied to the selected sample, and with confirmation coming from bibliography comparison, Chenomx NMR Suite 9.0, and Human Metabolome Database (HMDB) [32], as also reported elsewhere [33]. TOCSY experiments were carried out at 298 K using a spectral width of 15 ppm with direct and indirect dimensions with an 8 k × 256 data point matrix, a 3.00 s repetition time and 80 scans, while 80.00 ms was used for the spin-lock. The HSQC analyses were performed at 9.03 KHz (15 ppm) for the proton dimension and 30 KHz (200 ppm) for the carbon dimension, setting 8 k × 256 data point matrices for two dimensions, respectively, with 2 s of repetition delay and 96 scans. HMBC pulse sequences were acquired, employing spectral widths of 9.03 KHz (15 ppm) for the proton dimension and 37.5 KHz (250 ppm) for the carbon dimension, a data matrix of 8 k × 256 points, a repetition delay of 2 s, 96 scans and a long-range coupling constants ^1^H-^13^C of 8 Hz.

ACD Lab 1D-NMR Manager Ver. 12.0 software (Advanced Chemistry Development, Inc., Toronto, ON, Canada) was applied for the processing of one-dimensional NMR experiments, while JEOL Delta v5.3.1 software (JEOL Ltd., Tokyo, Japan) was used for two-dimensional experiments. All of the spectra were manually phased and baseline corrected using the baseline FID reconstruction protocol and referring to *TSP* (δ = 0.00).

*TSP* was also used as the quantitative internal standard, according to the following formula:

$$C_{m}=\frac{A_{m}}{A_{TSP}}\times \frac{H_{TSP}}{H_{m}}\times C_{TSP}$$

where *C_m_* is the concentration of the metabolite, *A_m_* is the area of the metabolite’s signal, *H_m_* is the number of protons related to the metabolite signal, *C_TSP_* is the *TSP* concentration, *A_TSP_* is the area of the *TSP* signal and *H_TSP_* is the number of protons related to the *TSP* signal.

Due to the urine dilution variability, the final concentration of all analytes was normalized to creatinine concentrations, whose diagnostic singlet was integrated at 4.05 ppm. The final concentration of metabolites was expressed as μmol/mmol of creatinine (Supplementary Table S2).

### 2.4. HPLC/Tandem Mass Spectrometry for Oxidative Stress Biomarkers Assessment

Urine samples were thawed in warm water, at about 37 °C [34], vortexed, and centrifuged at 10,000× *g* for 5 min; the internal standards were added to the urine supernatant which was then injected into the HPLC-MS/MS system. Samples were analyzed using a Series 200 LC quaternary pump (PerkinElmer, Norwalk, CT, USA) coupled with an AB/Sciex API 4000 triple quadrupole mass spectrometry detector, equipped with a Turbo Ion Spray (TIS) probe. The concentration of 8-oxoGua, 8-oxoGuo, and 8-oxodGuo was determined by means of isotopic dilution HPLC-MS/MS following the method described by Andreoli et al. [35], modifying some chromatographic conditions (solvents, chromatographic column, and mobile phases). The precursor/product ionic transitions monitored (in the positive ion mode) were 168.0 → 140.0 and 171.0 → 143.0 for 8-oxoGua and its internal standard ((13C15N2) 8-oxoGua), 284.3 → 168.0 and 287.13 → 171.1 for 8-oxodGuo and its internal standard ((13C15N2) 8-oxodGuo), 300.24 → 168.2 for 8-oxoGuo and 303.24 → 171.0 for its internal standard ((13C15N2) 8-oxoGuo), respectively. The monitored transitions of 3-NO_2_Tyr and its internal standard (3-NO_2_Tyr d3) were 226.99 → 181.0 and 229.99 → 184.0, respectively.

The limits of detection and quantification (LODs and LOQs) for oxidative stress biomarkers are 0.50 μg/L and 1.67 μg/L for 8-oxoGua; 0.14 μg/L and 0.48 μg/L for 8-oxodGuo; 0.70 μg/L and 2.33 μg/L for 8-oxoGuo; 1.81 μg/L and 6.03 μg/L for 3-NO_2_Tyr, respectively.

Analyst^®^ Software Version 1.5 (AB Sciex, Framingham, MA, USA) was employed for instrument control. The final concentration of the analytes was expressed in μg/g of creatinine to normalize values with respect to urine dilution variability. Urinary creatinine was determined by the method of Jaffè [36], using an alkaline picrate test with UV/Vis detection at 490 nm.

### 2.5. Chemical and Supplies

The analysis reference standard of 3-trymethylsilylpropionic acid 2,2,3,3-d4-acid sodium salt (TSP, degree of deuteration of 98%) was obtained from Sigma-Aldrich (Saint Louis, MO, USA), while that of deuterium oxide (D_2_O, degree of deuteration of 99.95%) was obtained from Sigma-Aldrich (Oakville, ON, Canada).

The pH was adjusted with sodium hydroxide (98% anhydrous granules, RPE for ACS-ISO analysis) and hydrochloric acid (30% ultrapure) obtained from Carlo Erba Reagents, Milan, Italy.

8-oxoGua, 8-oxodGuo and 8-oxoGuo analytical reference standards were obtained from Spectra 2000 SRL (Rome, Italy); 13C15N2-labeled isotopes of 8-oxodGuo and 8-oxoGuo were purchased from C/D/N Isotopes Inc. (Pointe-Claire, QC, Canada). The 13C15N2-labeled isotope of 8-oxoGua (98%) was purchased from Cambridge Isotope Laboratories Inc. (Tewkesbury, MA, USA). The analytical reference standard of 3-NO_2_Tyr was purchased from Cayman Chemical Company (Ann Arbor, MI, USA), while that of 3-NO_2_Tyr d3 was obtained from Toronto Research Chemicals (Toronto, ON, Canada).

### 2.6. Data analysis and Statistics

The NMR and HPLC-MS/MS combined data matrix was submitted to statistical multivariate analysis, considering thirty-four NMR metabolites and four HPLC-MS/MS oxidative stress biomarkers. Unsupervised PCA and supervised PLS-DA were applied to the entire dataset after centering and auto-scaling the entire dataset to highlight any differences between the two groups (exposed workers and not exposed) and between the workers before and after the working shift.

The PLS-DA model was validated by the leave-one-out cross validation method, using R^2^, which is the determination coefficient and is referred to as model fitting, and Q^2^, which is a prediction error measure, as model validation parameters. Significant variables for regression were selected based on regression coefficients, only taking into account those whose sign remained unchanged during the validation procedure [37,38].

Unscrambler 10.5 software (CAMO, Oslo, Norway) was employed for multivariate statistical analyses, while Sigmaplot 12.0 software (Systat Software, Inc., San Jose, CA, USA) was used for univariate analyses. Firstly, the Shapiro–Wilk test was employed to check the normality of the distribution, while parametric tests, such as Student’s *t*-test and the paired Student’s *t*-test, and non-parametric tests, such as the Mann–Whitney rank sum test and the Wilcoxon signed rank test, were applied for the identification of statistically significant variations in each variable between the groups analyzed. For statistical significance, a *p*-value of 0.05 was considered.

## 3. Results

**Figure 1:**
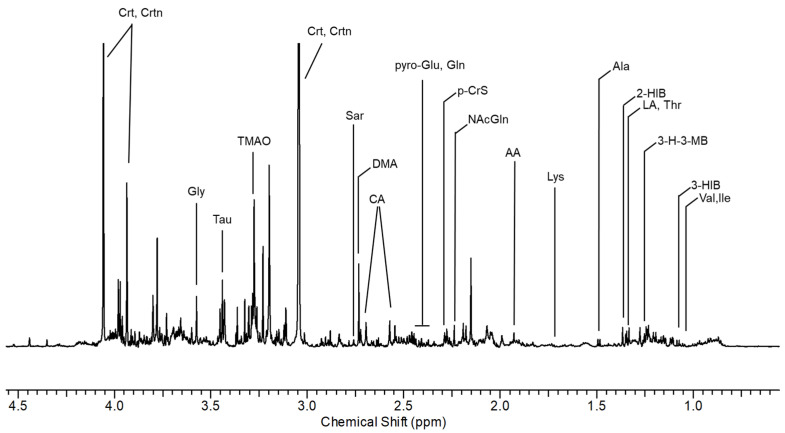
Spectral region of urine between 1.0 and 4.5 ppm from a shipyard worker at the end of the working shift. 2-HIB: 2-hydroxyisobutyric acid; 3-H-3-MB: 3-hydroxy-3-methylbutyric acid; 3-HIB: 3-hydroxyisobutyric acid; AA: acetic acid; Ala: alanine; CA: citric acid; Crt: creatine; DMA: dimethylamine; Gln: glutamine; Gly: glycine; Ile: isoleucine; LA: lactic acid; Lys: lysine; N-AcGln: N-acetylglutamine; p-CrS: p-cresol sulfate; pyroGlu: pyroglutamylglutamic acid; Sar: sarcosine; Tau: taurine; Thr: threonine; TMAO: trimethylamine n-oxide; Trig: trigonelline; Trp: triptophan; Tyr: tyrosine; U01: unknown 01; Val: valine.

**Figure 2:**
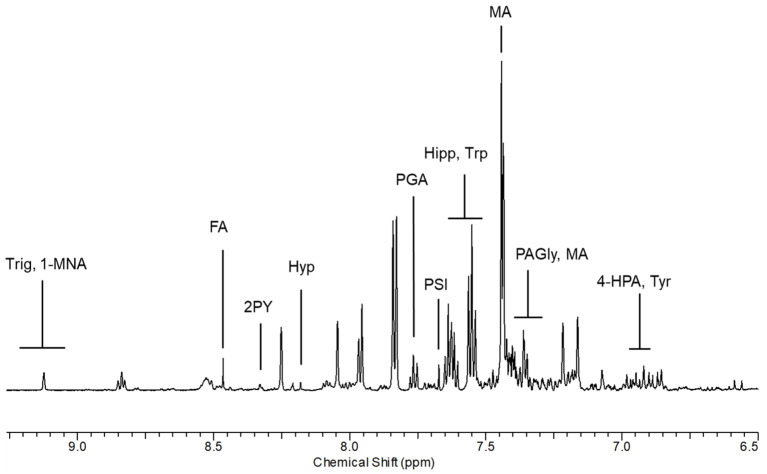
Spectral region of urine between 6.5 and 9.0 ppm from a shipyard worker at the end of the working shift. 1-MNA: 1-methylnicotinamide; 2PY: n-methyl-2-pyridone-5-carboxamide; 4-HPA: 4-hydroxyphenylacetic acid; FA: formic acid; Hipp: hippuric acid; Hyp; hypoxanthine; MA: mandelic acid; PAGly: phenylacetylglycine; PGA: phenylglyoxylic acid; PSI: pseudouridine.

Representative urinary ^1^H NMR spectra from AS workers with resonance assignment are shown in Figure 1 and Figure 2. Thirty-four urinary metabolites, excluding creatinine as a normalizing factor, were quantified from ^1^H NMR spectra of 17 healthy non-exposed controls and 17 shipyard workers, considered before (BS) and after (AS) the eight-hour work shift.

We choose to report and consider only those molecules whose resonances provided the optimum specificity and reproducibility.

From the comparison between non-exposed and AS exposed subjects, we observed qualitative differences in the spectral region between 7.40 and 7.97 ppm. From the analysis of the TOCSY spectrum, the correlation pattern of MA was identified, constituted by a multiplet between 7.40 and 7.43 ppm related to the aromatic protons and an intense signal at 7.44 ppm relating to the non-exchangeable protons of the hydroxyl groups. From the analysis of the one-dimensional spectrum, the presence of the alpha-proton was also observed, which appears as a singlet at 5.00 ppm. The monosubstituted aromatic system of PGA was then identified, whose correlations resonate at 7.61 (3,5-CH), 7.76 (4-CH) and 7.97 (2,6-CH) ppm.

The list of the identified metabolites containing different chemical classes of molecules such as amino acids, organic acids, and amines with the relative chemical shifts of the resonances are reported in Supplementary Table S1.

The NMR data matrix was integrated with a matrix of four oxidative stress biomarkers obtained from HPLC/MS-MS analyses: 8-oxoGua, 8-oxoGuo, 8-oxodGuo and 3-NO_2_Tyr. For the multivariate statistical analysis, the data were centered and autoscaled in order to make them comparable with each other in the final matrix.

Firstly, the unsupervised multivariate PCA analysis was conducted on 38 variables of the urine samples (n = 51) of subjects exposed (BS and AS) and not exposed (C) to styrene, in order to evaluate any spontaneous groupings between the samples. Supplementary Figure S1 shows the score plot for the PCA.

Even if the total variance explained is distributed over eight components, from the analysis of the first two components (29% of the total variance), a tendency towards separation along PC2 between all the samples of the exposed subjects (BS, AS) and C can be observed. Subsequently, further two PCAs were conducted, splitting BS and AS for the comparison with the C group.

As reported in Supplementary Figure S2A, no obvious grouping is observed between the BS subjects and the controls, which is mostly observable between the AS subjects and controls (Supplementary Figure S2B).

Based on the aforementioned results, we decided to separately analyze the data matrix by means of pairwise PLS-DA in order to identify the urinary metabolic profiles related to work shift (BS and AS) in respect to the control volunteer group.

**Figure 3:**
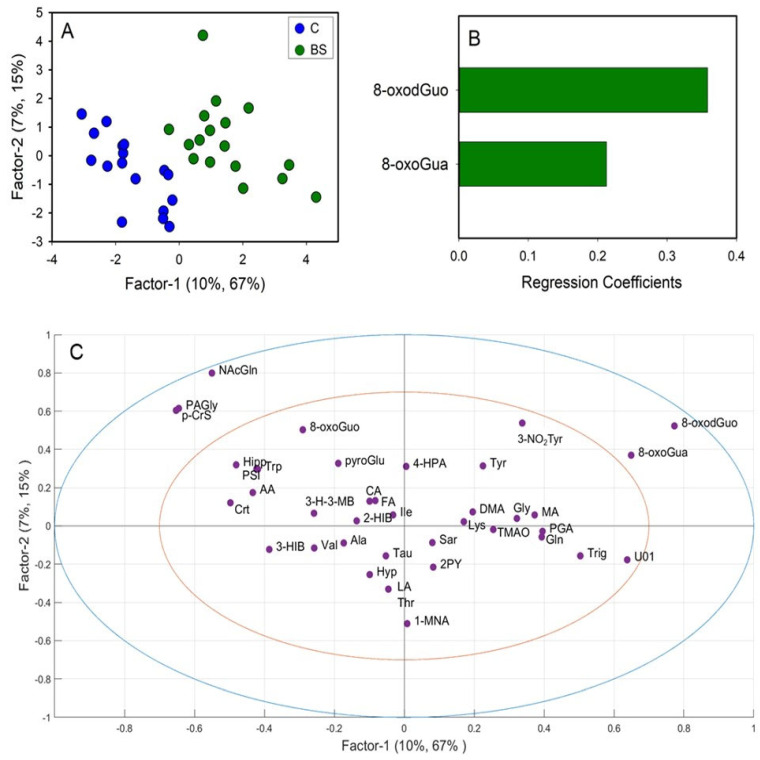
Partial least squares-discriminant analysis (PLS-DA) of integrated datasets of ^1^H-NMR and HPLC-MS/MS data, comparing controls (C) and workers before their shift (BS). (**A**) PLS-DA score plot displaying C (blue) and BS workers (green); (**B**) regression coefficient of the significant variables that discriminate BS (green) from C; (**C**) loadings plot. 1-MNA: 1-methylnicotinamide; 2-HIB: 2-hydroxyisobutyric acid; 2PY: N-methyl-2-pyridone-5-carboxamide; 3-H-3-MB: 3-hydroxy-3-methylbutyric acid; 3-HIB: 3-hydroxyisobutyric acid; 3-NO2Tyr: 3-nitrotyrosine; 4-HPA: 4-hydroxyphenylacetic acid; 8-oxodGuo: 8-oxo-7,8-dihydro-2′-deoxyguanosine; 8-oxoGua: 8-oxo-7,8-dihydroguanine; 8-oxoGuo: 8-oxo-7,8-dihydroguanosine; AA: acetic acid; Ala: alanine; CA: citric acid; Crt: crea-tine; DMA: dimethylamine; FA: formic acid; Gln: glutamine; Gly: glycine; Hipp: hippuric acid; Hyp; hypoxanthine; Ile: isoleucine; LA: lactic acid; Lys: lysine; MA: mandelic acid; N-AcGln: N-acetylglutamine; PAGly: phenylacetylglycine; p-CrS: p-cresol sulfate; PGA: phenylglyoxylic acid; PSI: pseudouridine; pyroGlu: pyroglutamylglutamic acid; Sar: sarcosine; Tau: taurine; Thr: threonine; TMAO: trimethylamine N-oxide; Trig: trigonelline; Trp: triptophan; Tyr: Tyrosine; U01: unknown 01; Val: valine.

**Figure 4:**
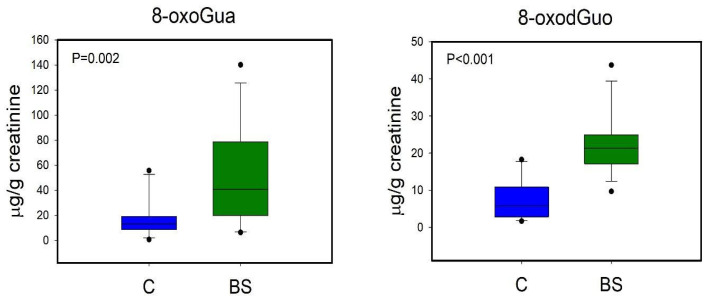
Boxplot of urinary metabolites that showed statistically significant changes between C (controls, blue) and BS workers (before shift, green). Statistical significance was assessed via the Mann–Whitney rank sum test. Boxplots report *p* values, median, minimum and maximum values (black dots), and the 25th and 75th percentile values of metabolites concentrations.

A first PLS-DA was carried out comparing the metabolic profiles of C and BS workers (Figure 3A). The PLS-DA model resulted in two significant latent variables (LV) and showed high robustness with R^2^ = 0.82 and Q^2^ = 0.52. Regression coefficient values (Figure 3B) showed that only two variables were important for the discrimination, specifically the levels of 8-oxoGua and 8-oxodGuo, which were higher in BS workers compared to C. Univariate analysis performed by via the Mann–Whitney rank sum test (*p* < 0.05) confirmed the significant differences in 8-oxoGua and 8-oxodGuo levels, being significantly higher in BS workers than in the controls (Figure 4).

**Figure 5:**
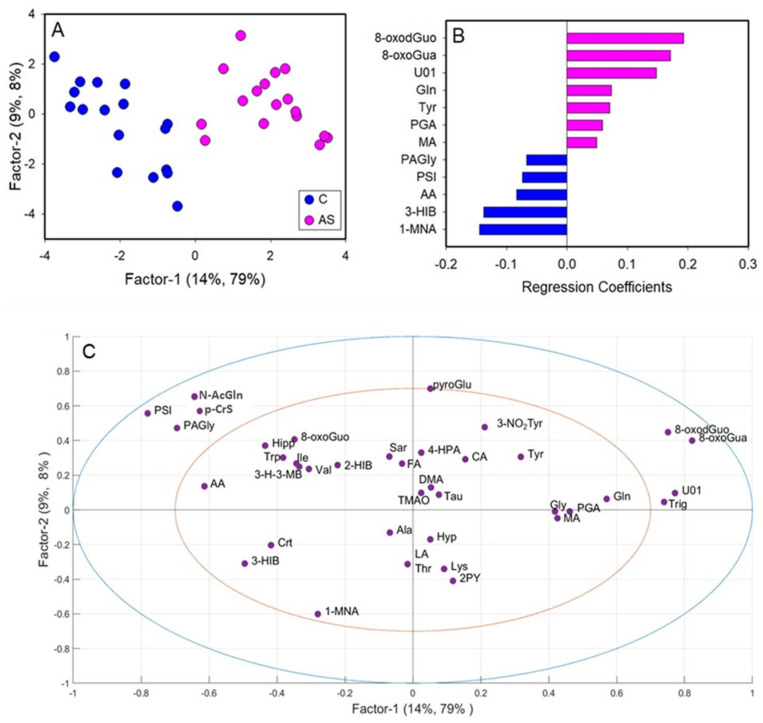
Partial least squares-discriminant analysis (PLS-DA) of integrated datasets of ^1^H-NMR and HPLC-MS/MS data, comparing controls (**C**) and workers after their shift (AS). (**A**) PLS-DA score plot displaying C (blue) and AS workers (magenta); (**B**) regression coefficient of the significant variables that discriminate BS (magenta) from C; (**C**) loadings plot. 1-MNA: 1-methylnicotinamide; 2-HIB: 2-hydroxyisobutyric acid; 2PY: N-methyl-2-pyridone-5-carboxamide; 3-H-3-MB: 3-hydroxy-3-methylbutyric acid; 3-hIB: 3-hydroxyisobutyric acid; 3-nO2Tyr: 3-nitrotyrosine; 4-HPA: 4-hydroxyphenylacetic acid; 8-oxodGuo: 8-oxo-7,8-dihydro-2′-deoxyguanosine; 8-oxoGua: 8-oxo-7,8-dihydroguanine; 8-oxoGuo: 8-oxo-7,8-dihydroguanosine; AA: acetic acid; Ala: alanine; CA: citric acid; Crt: creatine; DMA: dimethylamine; FA: formic acid; Gln: glutamine; Gly: glycine; Hipp: hippuric acid; Hyp; hypoxanthine; Ile: isoleucine; LA: lactic acid; Lys: lysine; MA: mandelic acid; N-AcGln: N-acetylglutamine; PAGly: phenylacetylglycine; p-CrS: p-cresol sulfate; PGA: phenylglyoxylic acid; PSI: pseudouridine; pyroGlu: pyroglutamylglutamic acid; Sar: sarcosine; Tau: taurine; Thr: threonine; TMAO: trimethylamine N-oxide; Trig: trigonelline; Trp: triptophan; Tyr: tyrosine; u01: unknown 01; Val: valine.

A second PLS-DA was performed on the data matrix comparing C and AS workers (Figure 5). The metabolic profiles of C and AS were effectively separated by inspecting the PLS-DA score plot (Figure 5A). The PLS-DA model showed two significant latent variables (LV) and a good model of discrimination, as reported by the R^2^ = 0.87 and Q^2^ = 0.70. Based on the regression coefficient values, twelve metabolites were identified as significantly relevant for the discrimination (Figure 5B). In particular, the levels of 3-hydroxyisobutyrate (3-HIB), acetate, phenylacetylglycine (PAGly), pseudouridine (PSI), and 1-methylnicotinamide (1-MNA) were lower in AS in respect to C, while the levels of glutamine (Gln), tyrosine (Tyr), MA, PGA, U01, 8-oxoGua and 8-oxodGuo were higher in AS workers.

**Figure 6:**
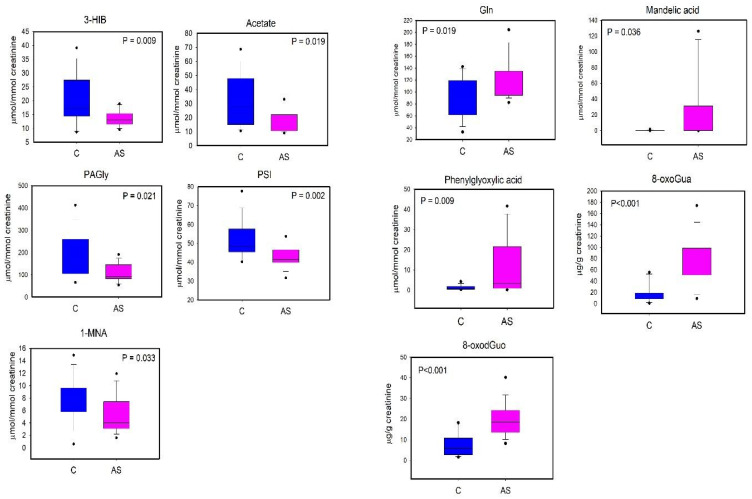
Boxplot of urinary metabolites that showed statistically significant changes between C (controls, blue) and AS workers (after shift, magenta). Statistical significance was assessed via the Mann–Whitney rank sum test. Boxplots report *p* values, median, minimum and maximum values (black dots), and the 25th and 75th percentile values of metabolites concentrations.

The differences in urinary 3-HIB, acetate, PAGly, PSI, 1-MNA, Gln, Tyr, MA, and PGA were tested for the univariate analysis (Student’s *t*-test or Mann–Whitney test according to the Shapiro–Wilk normality test). In Figure 6, we report the metabolites that showed a statistically significant difference (*p* < 0.05).

**Figure 7:**
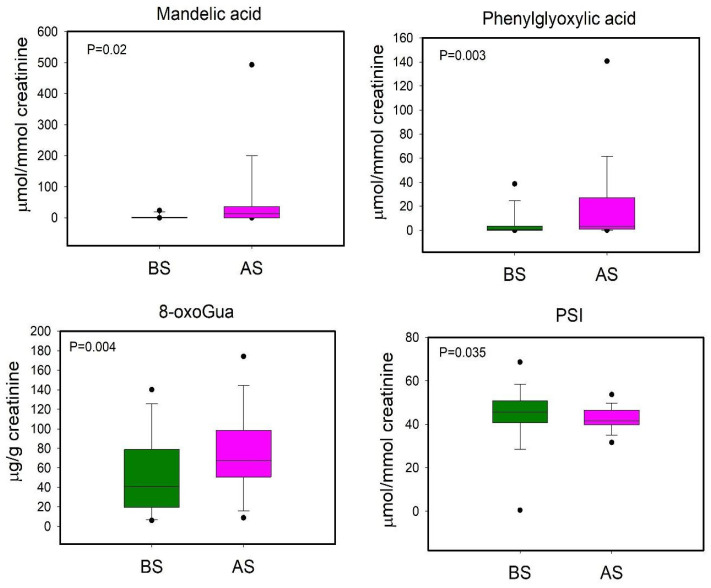
Boxplot plot of urinary metabolites that showed statistically significant changes between BS (before shift, green) and AS (after shift, magenta) workers. Statistical significance was assessed via the Wilcoxon signed rank Test. Boxplots report *p* values, median, minimum and maximum values (black dots), and the 25th and 75th percentile values of metabolites concentrations.

We performed a further PLS-DA in order to compare BS and AS workers, even though the model was not reliable (data not shown). Subsequently the univariate statistical analysis was applied to evaluate variations in metabolites’ urinary levels between before- and after-shift workers (Figure 7).

## 4. Discussion

Mandelic acid and phenylglyoxylic acid are the metabolic products of styrene oxide and are considered as urinary markers of exposure to styrene in humans [39]. Even though the results show that the urinary concentration of MA and PGA are far below the limit of 400 mg/g creatinine, as defined by ACGIH BEIs, in this work, we focused on the changes in the urinary metabolome in relation to MA and PGA concentrations, and hence to styrene exposure, since the styrene-derived metabolites, such as styrene oxide, could interact with DNA, RNA and proteins [33]. A correlation between the level of DNA damage and the excretion of mandelic acid and the concentration of styrene glycol in the blood has been shown, suggesting that DNA damage is the result of styrene exposure [40]. As shown in previous in vitro studies, the reaction between styrene oxide and DNA lead to adducts with dG, involving modification at the N-7, N^2^ and O^6^ positions, as well as adducts at the N^4^, N-3 and O^2^ positions of dC, the N-1 and N^6^ positions of dA and the N-3 position of T [41]. It has also been hypothesized that the same purine adducts can be generated on RNA. However, there is a lack of quantitative estimation of RNA adducts with styrene oxide [42]. Finally, styrene oxide can bind to proteins, such as hemoglobin and albumin [43]. Such conditions are strictly linked to an increase in the organism’s oxidative stress, which is the principal outcome of styrene exposure. In this context, the metabolic profiling of workers can provide a systemic overview of styrene occupational exposure.

Our results highlight that the urinary profile of exposed workers has more differences in respect to non-exposed ones, indicating both long- and short-term metabolic responses to styrene exposure.

As regards the long-term metabolic response to styrene exposure, we observed that BS workers displayed higher urinary levels of 8-oxoGua and 8-oxodGuo in respect to the non-exposed group (Figure 4). 8-oxoGua is usually associated with oxidatively generated damage to DNA and RNA, while 8-oxodGuo is the primary oxidation product of 2′-deoxyguanosine (dGuo) and is associated with damage to DNA [15]. This observation suggested a higher oxidative stress condition in workers already at the beginning of the work shift, as observed in other studies regarding oxidative stress in occupational settings [33].

As regards the short-term metabolic response to styrene exposure, our results showed increased levels of MA, PGA and 8-oxoGua in AS workers and decreased levels of PSI in respect to the samples taken before the shift. Comparing BS and AS workers, we suggest that these metabolites could define the urinary profile related to the short-term response to styrene exposure. PSI is a post-transcriptional RNA modification and is the most abundant modified nucleoside in RNA [44], and it is excreted in urine without further modifications as the final product of RNA catabolism [45]. Its urinary levels reflect RNA turnover and, indirectly, protein turnover [46,47].

The same subjects after their shift showed a more complex urinary profile compared to the non-exposed subjects. PSI, 1-MNA, 3-HIB, acetate, and PAGly were lower in AS than in the non-exposed group. This observation could be associated with a metabolic response to the increased oxidative stress condition in AS workers, due to the acute exposure during the work shift, even if the workers wore personal protective equipment.

1-MNA is produced by the transformation of NAM by the enzyme nicotinamide-N-methyl transferase (NNMT), and it is a catabolic intermediate of nicotinamide-adenine-dinucleotide (NAD). NAD is not only required for key metabolic pathways, but it is also essential for DNA repair mechanisms [48]. We can speculate that a higher turnover of nicotinamide for the NAD synthesis could occur in exposed workers, involving the NAD salvage pathway, where NAD^+^ is generated by transforming nicotinamide into nicotinamide mononucleotide (NMN) [49]. These findings could suggest a higher metabolic response for restoring NAD^+^/NADH balance in response to alterations of the oxidative state at the end of the working shift.

3-HIB, acetate, PAGly and Gln can be considered as comprising an altered aminoacidic pathway occurring at the end of the work shift, after eight hours of low-dose exposure. 3-HIB is the direct catabolite of valine [50], and its lower levels in AS workers could suggest a reduced hepatic metabolism of valine after acute exposure to styrene.

PAGly and acetate are exclusive products of bacterial phenylalanine metabolism [51,52]. Workers displayed higher levels of 8-oxoGua as an index of higher oxidative stress conditions after styrene exposure, and a correlation between oxidative stress and gut microbiota alterations has been widely assessed [53,54,55]. We can speculate that the lower levels of PAGly and acetate in AS workers compared to the non-exposed group could suggest an altered gut microbiota following styrene exposure. Given the lack of studies regarding the correlation between occupational exposure and the gut microbiota, this observation remains speculative and should be supported by future investigations.

Gln is an amino acid involved in urea synthesis, ammoniagenesis, and gluconeogenesis, and is a major respiratory fuel for many cells [56,57]. It has been shown that chronic exposure to styrene at levels below the TLV–TWA induced IL-6 and TNFα expression, which are involved in the regulation of several pathways in liver regeneration after hepatotoxic exposure [58]. The intestinal ammonia, produced from nitrogen products, is taken up by the liver and metabolized into urea. Previous studies have shown that styrene or its metabolites can induce direct hepatotoxicity by covalently binding to intracellular molecules or via oxidative stress [59,60], and an excessive production of reactive oxygen species (ROS) affects liver cellular function. During short-term exposure below the TLV–TWA, ammonia could accumulate through the temporary reduction in the hepatocyte functionality and, in this context, the increased levels of Gln observed could be due to the persistently increased activity of hepatic Gln synthase, which catalyzes the condensation of ammonia with glutamate to produce Gln, contributing to ammonia detoxification.

## 5. Conclusions

For the first time, we investigated, via a metabolomic multiplatform approach, the urinary metabolic profile of shipyard workers exposed to styrene vapors and the differences in the urinary metabolic profiles of workers exposed to styrene compared to a non-exposed group, as well as the differences in the profiles of the same subjects sampled before and after their work shift.

From the multivariate and univariate analyses, the urinary metabolites most involved in the exposure to styrene were identified and associated with a systemic perturbation, and intriguing findings have been highlighted. From the comparison between C and BS workers, long-term effects due to the exposure have been found, with higher levels of 8-oxoGua and 8-oxodGuo. This result implies the need to further investigate such long-term effects occurring due to the reiterative exposure to styrene, even at levels below the TLV–TWA.

Comparisons between C and AS, as well as comparisons between BS and AS workers, showed as short-term effects (after eight-hour working shift) higher levels of styrene biomarkers associated with alterations to urinary metabolites linked to an oxidative stress condition (PSI, 1-MNA, Gln) and gut microbiota activity (PAGly, acetate).

In addition, it should be noted that in the shipyard working environment, exposure to multiple xenobiotics can occur, including wood dust and other volatile organic compounds, which should be better investigated in further studies and correlated with clinical outcomes.

## Figures and Tables

**Table 1:** Characteristics of the investigated subjects.

	Age (Mean ± SD)	Males (N)	Females (N)	Smokers (N)	Alcohol Consumption (N)
Controls	59.4 ± 4.8	17	0	0	0
Workers	45.8 ± 7.1	17	0	6	0

## Data Availability

Data are available in the Supplementary Materials.

## Supplementary Materials

The following supporting information can be downloaded at: https://www.mdpi.com/article/10.3390/toxics12030182/s1, Figure S1: Score plot for PCA performed on non-exposed subjects (C, blue) and exposed BS (green) and AS (magenta) subjects; Figure S2: PCA scores plots performed on A) BS subjects (green) and C (blue) and B) AS subjects (magenta) and C (blue); Table S1: ^1^H chemical shifts of metabolite signals in urine samples; Table S2: Urinary concentrations of metabolites.
