# Supplementary material for: NMR Untargeted and HPLC-MS/MS Targeted Metabolomic Approaches for Evaluating Styrene Exposure in the Urine of Shipyard Workers
Source: Toxics. 2024 Feb 28;12(3):182. doi: 10.3390/toxics12030182 (PMC10975827; doi:10.3390/toxics12030182)
Supplement: Supplementary file 1 [file toxics-12-00182-s001.zip › supplementary/Supplementary.pdf]

NMR untargeted and HPLC-MS/MS targeted metabolomic approaches for evaluating styrene exposure in the urine of shipyard workers

Ottavia Giampaoli[a,b], Fabio Sciubba[a,b], Giovanna Tranfo[c], Renata Sisto[c], Daniela Pigini[c], Michele De Rosa[d], Adriano Patriarca[d], Alfredo Miccheli[a,b], Anna Rita Fetoni[e], Laura Tricarico[f] and Mariangela Spagnoli[c,*]

[a] *NMR-based Metabolomics Laboratory (NMLab), Sapienza University of Rome, Piazzale Aldo Moro 5, 00185, Rome, Italy;*

[b] *Department of Environmental Biology, Sapienza University of Rome, Piazzale Aldo Moro 5, 00185, Rome, Italy;*

[c] *Department of Occupational and Environmental Medicine, Epidemiology and Hygiene, INAIL, Via Fontana Candida 1, 0078, Monte Porzio Catone, Italy;*

[d] *Department of Chemistry, Sapienza University of Rome, Piazzale Aldo Moro 5, 00185, Rome, Italy*

[e] *Department of Neuroscience, Reproductive and Odontostomatological Sciences-Audiology Section, University of Naples Federico II, 80131 Naples, Italy;*

[f] *Catholic University of the Sacred Hearth, Faculty of Medicine and Surgery, Largo Agostino Gemelli 8, 00168, Rome, Italy;*

***Corresponding Author:** Dr. Mariangela Spagnoli, Department of Occupational and Environmental Medicine, Epidemiology and Hygiene, INAIL, Via Fontana Candida 1, 0078, Monte Porzio Catone, Italy, m.spagnoli@inail.it

Keywords: styrene exposure; NMR-based metabolomics; oxidative stress biomarkers; urinary metabolic profiles

Supplementary Material

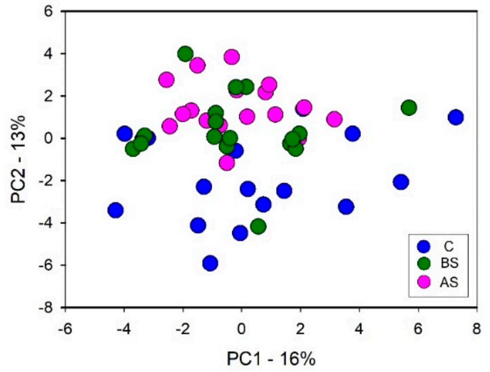

Supplementary Figure S1. Scores plot for PCA performed on non-exposed subjects (C, blue) and exposed subjects BS (green), AS (magenta). Even if the total variance explained is distributed over 8 components, from the analysis of the first two components (29% of the total variance) a tendency towards separation along PC2 between all the samples of subject exposed (BS, AS) and C can be observed.

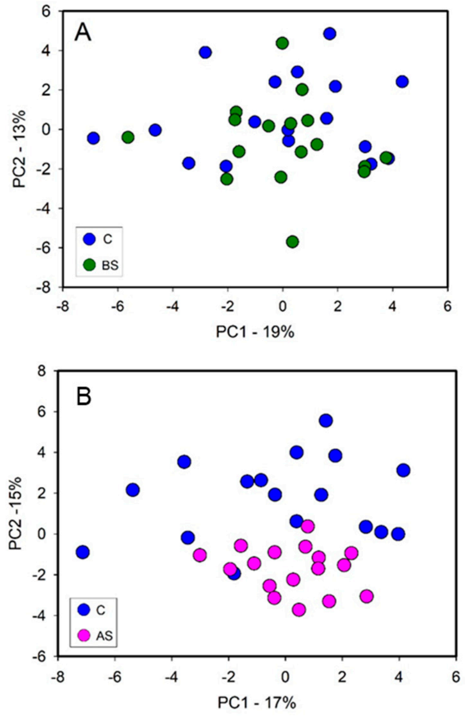

Supplementary Figure S2. PCA scores plots performed on A) subjects BS (green) and C (blue), B) subjects AS (magenta) and C (blue). No obvious groupings are observed between BS subjects and controls, which is mostly observable between AS subjects and controls.

Supplementary Table 1. [1]H chemical shifts of metabolite signals in urines. The resonances choose for the quantification are reported in bold.

bs: broad singlet; d: doublet; dd: doublet of doublets; m: multiplet; q: quadruplet; s: singlet; t: triplet.

Metabolite	Structure	^{1}H δ ppm	Multiplicity	Assignment
1. Valine (Val)	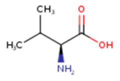	0.99<br>**1.05**	d<br>**d**	CH_3<br>**CH_3'**
2. Isoleucine (Ile)	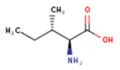	0.92<br>**1.01**<br>1.99	t<br>**d**	CH_3<br>**CH_3**<br>CH
3. 3-Hydroxyisobutyrate (3-HIB)	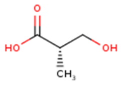	**1.07**<br>2.49<br>3.54<br>3.71	**d**	**CH_3**
4. 3-Hydroxy-3-methylbutyrate (3-H-3-MB)	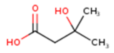	**1.27**	**s**	**CH_3, CH_3'**
5. Lactate	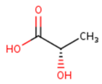	**1.33**<br>4.11	**d**<br>q	**CH_3**<br>CH
6. Threonine (Thr)	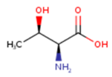	**1.33**<br>3.59<br>4.26	**d**<br>d<br>m	**CH_3**<br>α-CH<br>β-CH
7. 2-Hydroxyisobutyrate (2-HIB)	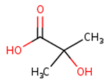	**1.36**	**s**	**CH_3, CH_3'**
8. Alanine (Ala)	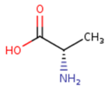	**1.49**<br>3.78	**d**<br>q	**CH_3**<br>α-CH
9. Lysine (Lys)	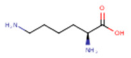	1.48<br>**1.71**<br>1.89<br>3.02<br>3.74	m<br>**m**<br>m<br>m<br>m	CH_2<br>**CH_2**<br>CH_2<br>CH_2<br>CH
10. N-acetylglutamine (NAcGln)	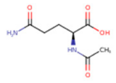	1.95<br>2.12<br>**2.27**<br>4.18<br>7.97	**m**<br>bs	**CH_2**
11. p-Cresol sulphate	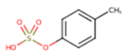	**2.35**<br>7.21<br>7.28	**bs**<br>dd<br>dd	**CH_3**<br>CH, CH'<br>CH, CH'

12. Pyroglutamate (pyro-Glu)	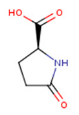	2.03<br>**2.40**<br>2.50<br>4.19	m<br>**m**<br>m<br>dd	CH_2<br>**CH**<br>CH<br>CH
13. Glutamine (Gln)	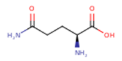	2.13<br>**2.46**<br>3.78	m<br>**m**<br>t	CH_2<br>**CH_2**<br>CH
14. Citrate	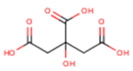	**2.54**<br>2.69	**d**<br>d	**CH, CH’**<br>CH, CH’
15. Dimethylamine (DMA)	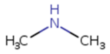	**2.73**	**s**	**CH_3, CH_3’**
16. Sarcosine (Sar)	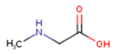	**2.78**	**s**	**CH_3**
17. Creatine	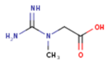	**3.05**<br>3.95	**s**<br>s	**CH_3**<br>CH_2
18. Creatinine	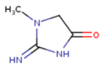	3.03<br>**4.05**	s<br>**s**	CH_3<br>**CH_2**
19. Trimethylamine N-Oxide (TMAO)	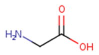	**3.27**	**s**	**CH_3**<br>CH_2
20. Taurine (Tau)	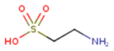	3.27<br>**3.43**	t<br>**t**	CH2<br>**CH2**
21. Glycine (Gly)	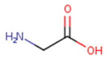	**3.57**	**s**	**CH_2**
22. 4-Hydroxyphenylacetate (4-HPA)	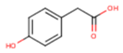	**6.87**<br>7.17	**dd**<br>dd	**CH,CH**<br>CH,CH
23. Tyrosine (Tyr)	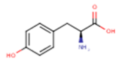	**6.90**<br>7.18	**dd**<br>dd	**2-CH,6-CH**<br>3-CH,5-CH
24. 4-Hydroxybenzoate (4-HBz)	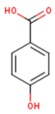	6.97<br>**7.76**	dd<br>**dd**	2-CH,6-CH<br>**3-CH,5-CH**
25. Tryptophan (Trp)	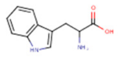	7.20<br>7.27<br>7.29<br>**7.50**<br>7.70	m<br>m<br>bs<br>**d**<br>d	5-CH<br>6-CH<br>2-CH<br>**4-CH**<br>7-CH
26. Phenylacetylglycine (PAGly)	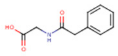	**7.36**<br>7.42	**m**<br>m	**2,4,6-CH**<br>3,5-CH

27. Mandelic acid (MA)	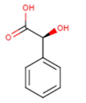	5.00<br>7.38-7.43<br>7.42	s<br>m<br>**s,s**	CH<br>2-5-CH<br>OH, OH’
28. Hippurate (Hipp)	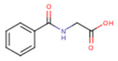	3.97<br>**7.55**<br>7.64<br>7.83	d<br>**m**<br>m<br>m	CH_2<br>**3,5-CH**<br>4-CH<br>2,6-CH
29. Pseudouridine (PSI)	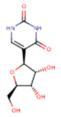	**7.67**	**bs**	**CH**
30. Phenylglyoxylic acid (PGA)	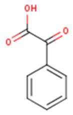	7.60<br>**7.76**<br>7.96	m<br>m<br>m	5,3-CH<br>**4-CH**<br>6,2-CH
31. Hypoxanthine (Hyp)	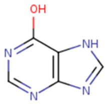	**8.19**<br>8.21	s<br>s	2-CH<br>7-CH
32. N1-Methyl-2-pyridone-5-carboxamide (2PY)	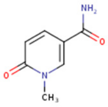	3.64<br>6.67<br>7.98<br>**8.33**	s<br>d<br>d<br>**dd**	N-CH_3<br>3-CH<br>4-CH<br>**6-CH**
33. Formate	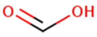	**8.46**	**s**	**CH**
34. U01(2-Methylnicotinamide?)		8.06<br>8.54<br>**8.78**	**d**	**6-CH**
35. Trigonelline (Trig)	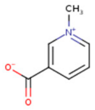	4.44<br>8.08<br>8.84<br>**9.12**	s<br>m<br>m<br>**s**	N-CH_3<br>5-CH<br>4,6-CH<br>**2-CH**
36. 1-Methylnicotinamide (1-MNA)	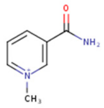	8.17<br>8.89<br>8.96<br>**9.28**	t<br>d<br>d<br>**s**	5-CH<br>4-CH<br>6-CH<br>**2-CH**
